# Patient-Reported Outcomes Following Sectioning of the Filum Terminale for Treatment of Tethered Cord Syndrome Associated With Ehlers-Danlos Syndrome

**DOI:** 10.7759/cureus.24679

**Published:** 2022-05-02

**Authors:** Alissa Zingman, Kelly Tuchman, Fraser Henderson, Clair A Francomano

**Affiliations:** 1 Preventive Medicine, PRISM Spine and Joint, Bethesda, USA; 2 Neurological Surgery, Metropolitan Neurosurgery Group, Bethesda, USA; 3 Neurological Surgery, University of Maryland Capital Region Medical Center, Largo, USA; 4 Neurological Surgery, The Metropolitan Neurosurgery Group, Bethesda, USA; 5 Department of Medical and Molecular Genetics, Indiana University School of Medicine, Indianapolis, USA

**Keywords:** back pain, filum terminale, hypermobility disorder, ehlers danlos syndrome, tethered cord syndrome

## Abstract

Introduction

Tethered cord syndrome (TCS) was first reported as a potential complication of Ehlers-Danlos Syndrome in 2009. However, there have been few publications on the subject since that time, and optimal treatment of TCS in the setting of the hypermobile Ehlers-Danlos Syndrome (hEDS) population remains unknown. The purpose of this study was to determine the safety and efficacy of surgical release of the filum terminale (FT) for the treatment of TCS in this patient population.

Methods

We performed a retrospective chart review of consecutive hEDS patients with TCS who were treated with surgical release after providing informed surgical consent over a 4.5-year period by a single neurosurgeon. Eighty-four patients were identified and asked to complete surveys with items regarding pre and postoperative symptoms, pain levels, and satisfaction.

Results

Thirty patients with a mean age of 30.8 ± 11.9 years, all female, were included. Low back pain was significantly improved across the entire cohort. For patients with both pre and postoperative data available, the distance they were able to walk also improved significantly. The majority of patients were “highly satisfied” with surgery (66%), followed by 21% “satisfied”, 10% “neutral”, and one patient who was “dissatisfied”. One patient required repair of a dural leak one week postoperatively, and no other complications were noted.

Conclusions

Surgical release of the FT for TCS in patients with hEDS was safe and effective in this cohort. For most patients, there was a significant improvement in low back pain, urinary symptoms, and ability to ambulate distance. The majority of respondents reported subjective satisfaction with this operation. A further prospective study is warranted.

## Introduction

The Ehlers Danlos Syndrome (EDS) is a heterogeneous group of heritable connective tissue disorders characterized by joint hypermobility, skin extensibility, and tissue fragility [1-2]. Fourteen types of EDS have been described, of which hypermobile EDS (hEDS) is the most common [1]. Tethered cord syndrome (TCS) is a disorder characterized by neurological, urological, orthopedic, and radiological findings, attributable in part to a thickened or taut filum terminale (FT), usually in association with a low-lying conus medullaris (CMD) [3]. The seminal contribution of Warder and Oakes demonstrated that the conus could be in the normal position and yet be considered tethered [4]. Their term tethered cord syndrome with normally positioned conus has subsequently been supplanted by the term occult TCS [5]. Regardless of the conus position, release of the FT is a well-accepted surgical treatment for TCS [4-10].

TCS has been associated with hEDS, and most patients have the conus in the normal position [2,6-7]. These patients do not generally have a Chiari malformation, though spina bifida occulta is frequently present. Though the underlying pathophysiology of TCS remains controversial, recent studies have reported on the presence of inflammatory cells and decreased elasticity of the filum of EDS patients - findings that are congruent with an underlying biomechanical stretch mechanism as proposed by Yamada [3,11]. Few studies have reported on the surgical outcomes of hEDS patients who undergo sectioning of the filum [6,11]. The goal of this pilot study was to assess whether sectioning of the filum terminale in subjects with a clinical diagnosis of TCS and hEDS is safe and efficacious in terms of relief of pain and improvement of neurological and urinary bladder symptoms.

## Materials and methods

This study was approved by the Ethics Review Board at Greater Baltimore Medical Center. We retrospectively queried the database for patients that underwent surgery for TCS from June 2010 to December 2014. All included subjects carried a diagnosis of the hypermobility type of EDS, based on the Villefranche criteria for diagnosis of EDS [12], and all met the following inclusion and exclusion criteria for TCS surgery.

Inclusion criteria: 1. Severe low back and leg pain; 2. Symptoms of urological dysfunction, including urinary frequency, hesitancy, urinary tract infection, nocturia, incomplete emptying, and incontinence; 3. Weakness and sensory loss of the lower extremities; 4. Positive stretch or traction sign.

Exclusion criteria: 1. Demonstration of instability, clinically significant disc herniation, and stenosis or severe osteoarthritic changes of the cervical, thoracic or lumbar spine that would reasonably explain the pain, weakness, and sensory changes of the lower extremities and urinary bladder dysfunction; 2. Spinal neoplasm: 3. Neuropathy due to diabetes and other metabolic conditions, nutritional deficiency, or genetic disorders such as Charcot-Marie-Tooth; 4. History of traumatic spinal cord injury; 5. Inflammatory, infectious, or degenerative connective tissue disorders such as neuro-borreliosis, ankylosing spondylitis, or rheumatoid arthritis.

Eighty-four patients who met the above surgical criteria and had undergone sectioning of the filum were sent a survey via e-mail or fax. Clinical notes and operative reports were reviewed by a research nurse to determine patients’ prior history with particular attention placed on their neurological and urological findings. Urodynamic studies were also reviewed to characterize bladder function. Determination of the conus position was made by MRI on sagittal T2-weighted images. A conus residing above the mid-L2 was considered the “normal position.” In the setting of a clinical diagnosis of TCS, a normal position of the conus would confer the term occult.

Outcome measures: 1. Visual analog scale (VAS) for low back pain; 2. Symptom questionnaire (pre and postoperative); 3. Patient satisfaction scores (ordinal):

0 = dissatisfied

1 = neutral (neither satisfied nor dissatisfied)

2 = somewhat satisfied

3 = highly satisfied

Surgical technique and postoperative protocol

**Figure 1:**
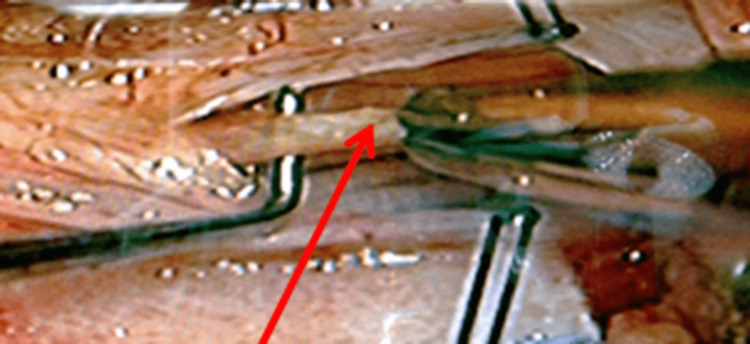
Intraoperative visualization of the filum terminale (red arrow) prior to cauterization and sectioning

All surgeries were performed by one surgeon at two hospitals. With the patient prone, an S1 laminectomy was performed. The lamina was kept in cold saline for later replacement. A midline durotomy was performed. The arachnoid was opened and clipped to the edge of the dura. Microsurgical mapping of the cauda equina with sacral nerve electrophysiological stimulation was employed in every case to confirm the identification of the FT (Figure 1). This also enabled the identification of the sacral nerve fibers adherent to the filum, which could then be dissected free. The threshold of stimulation of the FT ranged from 3 mA to 7 mA - or approximately three times the threshold demonstrated for sensory nerves.

A 1-cm portion of the FT was cauterized at low voltage, sectioned, and retained as a pathological specimen. The dura was closed with 6-0 Prolene sutures, and a Valsalva maneuver was performed to confirm water-tight closure. A laminoplasty was performed by replacing the lamina with titanium miniplates (Depuy Synthes, Raynham, MA), 6 mm screws, and demineralized bone. The wound was closed in layers, and the patient was kept on strict bed rest for 48 hours, after which they were granted bathroom privileges. Patients were usually discharged on the third postoperative day.

Data analysis

Bivariate analysis was performed using the paired t-test to compare pre and postoperative continuous variables. χ2 and Fisher’s exact tests were used to compare categorical variables between pre and postoperative outcomes. Sub-analysis was performed to determine if outcomes were dependent on whether the TCS could be identified on imaging preoperatively versus occult cases.

Although patient satisfaction was collected as an ordinal variable (0, 1, 2, 3), due to the small number of patients in the first two bins, 0 and 1 were collapsed into “not satisfied” and 2 and 3 into “satisfied” bins. Potential predictors of patient satisfaction were first tested in bivariate analysis, and those variables found to be associated (P ≤ 0.10) with patient satisfaction in bivariate testing were used in a forward stepwise multivariate binary logistic regression analysis. Odds ratios with 95% confidence intervals (CIs) were determined for all significant predictors, and model fit was confirmed using the Hosmer-Lemeshow test. All statistical analysis was performed using Minitab for Windows, version 20.3 (Minitab LLC, State College, PA).

## Results

Demographics and preoperative characteristics

Thirty patients completed surveys, for a response rate of 35.7%. All patients were female, with a mean age of 30.8 ± 11.9 years (range 15-60). The mean follow-up interval was 16.9 ± 12.9 months. Five patients had undergone previous untethering of the filum terminale. Preoperative urodynamics confirmed neurogenic bladder in 24 (80%) patients. Fifteen of 30 patients had preoperative MRI findings consistent with TCS while the remaining 15 cases were rated as occult.

Postoperative outcomes

**Figure 2:**
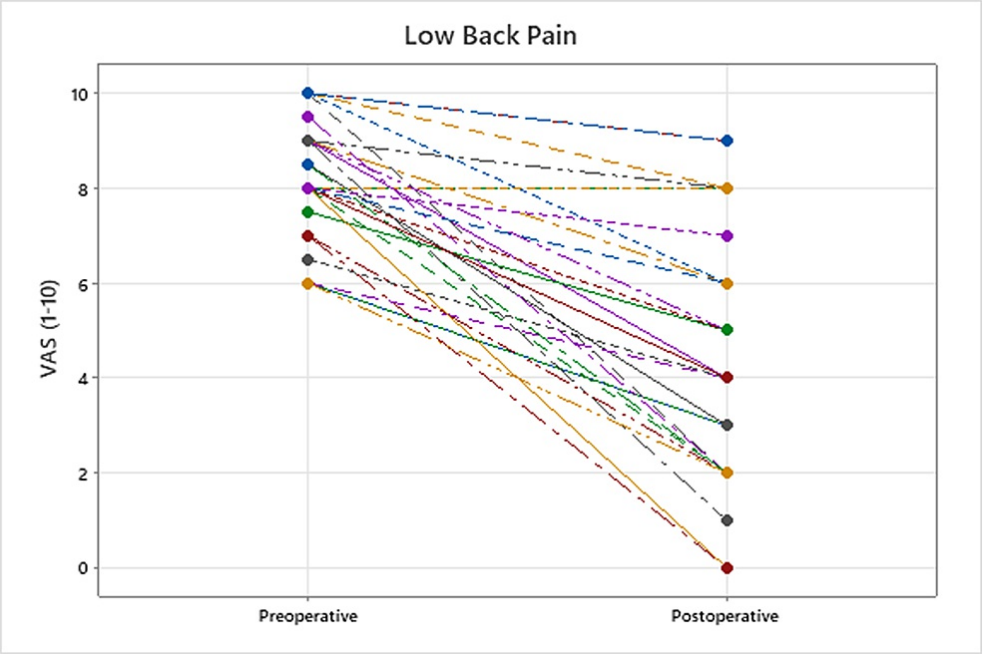
Change after surgery in VAS low back pain for all patients VAS: visual analog scale

Low back pain was significantly improved across the cohort (mean preoperative vs. postoperative VAS 8.2 ± 1.3, vs. 4.3 ± 2.6; P < 0.001; Figure 2). For 19 patients with both pre and postoperative data available, the distance they were able to walk also improved significantly (mean preoperative vs. postoperative distance 359 ± 438 feet, vs. 3743 ± 3607 feet; P = 0.003).

After low back pain, the non-urinary-related symptoms that were most often improved postoperatively were leg weakness (77%), lower extremity sensory loss (40%), and pain with stairs (30%). Among urinary symptoms, incontinence (40%) and frequency (33%) were improved in the largest proportion of patients and were not worsened in any. While nocturia (33%) and difficulty emptying the bladder (30%) were also improved in most patients, worsening postoperatively was reported by 17%. Occult versus radiographic TCS on preoperative MRI had no significant effect on any outcome studied. Categorical outcomes are presented (Table 1).

**Table 1:** Presence and change in frequency of symptoms among participants NS = Not significant; LE = lower extremity; UTI = urinary tract infection

Symptom	Improved Postoperatively (%)	Worsened Postoperatively (%)	No Change Postoperatively (%)	P-value
Low Back Pain	90	3	7	<0.001
Tripping	27	3	70	0.031
Leg Weakness	77	0	23	0.006
Pain with Stairs	30	0	70	0.001
LE Sensory Loss	40	0	60	0.001
LE Cramps	7	13	80	NS
Recurrent UTIs	27	10	63	NS
Nocturia	33	17	50	NS
Difficulty Emptying Bladder	30	17	53	1.0
Urinary Urgency	20	3	77	0.004
Urinary Frequency	30	0	70	0.023
Urinary Incontinence	66	0	60	<0.001
Bowel Incontinence	13	10	77	0.120

Complications and additional surgeries

One patient required surgical repair of a dural leak approximately one week after surgery but subsequently recovered well. The leak occurred when the patient hyper-flexed her lumbosacral junction one week after the surgery, causing a tear in the durotomy. Another patient underwent lumbar surgery (L4-5 fusion) 16 months after the tethered cord surgery, and one underwent a sacroiliac fusion five years later. 

Satisfaction scores

**Figure 3:**
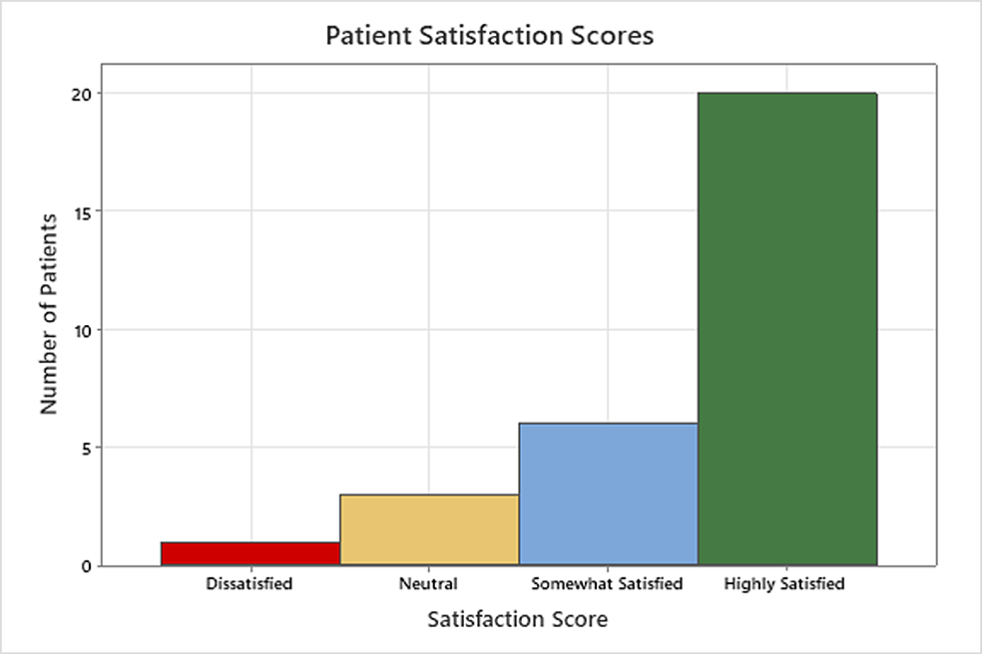
Patient satisfaction

The majority of patients (87%) were satisfied with the surgery (66% “highly”, 21% “somewhat”) (Figure 3). Three (10%) patients were neutral (neither satisfied nor dissatisfied), two of whom underwent further lumbar or sacroiliac fusion, and a third reported initial relief but then return of pain one year postoperatively. A single patient expressed dissatisfaction, reporting slight improvement in low back pain and leg numbness, but no improvement in urinary symptoms, and rather increasingly frequent urinary tract infections postoperatively.

When asked if they would undergo surgery again, one patient replied “no” and another was unsure; the remaining 28 patients would undergo the surgery again. In bivariate analysis, change in low back pain VAS was found to be a significant predictor of satisfaction (median change in VAS = 4; 95% CI, 3 to 5.7 satisfied vs. VAS change = 1; 95% CI, 0 to 2 not satisfied; Mood’s median test: P = 0.032 ). Improvement in weakness was also a significant predictor of patient satisfaction (Fisher’s exact test; P = 0.003). The final logistic model was found to be significant (P < 0 .047) and correctly predicted 93% of outcomes. In the final logistic regression model, only the change in VAS for low back pain was associated with patient satisfaction (odds ratio = 3.56, 95% CI, 1.02 to 12.44; P < 0 .047).

## Discussion

EDS is a heterogeneous group of heritable connective tissue disorders most notably characterized by the combination of joint hypermobility, pain, and generalized tissue fragility [2]. In 2017, the EDS International Consortium created a new international classification describing 13 different subtypes of EDS, of which the hypermobile type (hEDS) is the most prevalent. While molecular testing is used to identify the 12 other variants of EDS, diagnosis of hEDS is currently based solely upon clinical findings [1]. The patients described in this study were assessed prior to 2017 and hence were diagnosed according to the Villefranche criteria, diagnostic criteria preceding the 2017 criteria [12].

Patients under consideration for tethered cord release should undergo a rigorous diagnostic to reasonably exclude other conditions. TCS as a clinical diagnosis is characterized by neurological and urological findings and often orthopedic, cutaneous, and radiological findings. Neurological symptoms include aching or burning pain in the low back and leg, worse when walking upstairs or on an inclined plane; paresthesias in the pelvic area or lower extremities, sensory loss in the lumbar and sacral dermatomes, and leg weakness, heaviness, and stiffness which is often asymmetric. Urogenital findings include urgency, frequency, incontinence, large bladder, incomplete emptying, nocturia, irregular urinary stream, frequent urinary tract infections (>3 per year), and sexual dysfunction. Fecal incontinence and constipation occur less commonly. The neurological exam shows weakness, sensory loss in the lumbar and sacral dermatomes, a positive stretch/traction sign, and hyper or hyporeflexia. Orthopedic findings include scoliosis, kyphosis, pes planus, hammertoes, varus deformity, and ankle deformities. Cutaneous findings are more common in the early pediatric group than in the adult group and consist of sacral dimple, lipoma, hairy patch, and hemangioma. Radiological findings may include a low-lying CMD, a thickened FT (> 2mm), or evidence of abnormal tissue, such as fatty infiltration.

While previous studies have distinguished between TCS and what is termed occult TCS [13], i.e. TCS with the normal position of the conus, Warder and Oakes noted no difference in surgical outcome measures between those subjects in whom the MRI demonstrated the conus at the normal position (the occult TCS subjects) and those in whom the conus was demonstrated below the L2 level [4,14].

Surgical release of the FT is a well-accepted treatment for TCS [15]. In a series of 60 children with occult TCS, Wehby et al. reported significant improvement in urinary symptoms, fecal incontinence, pain, and sensorimotor deficiencies with the release of the FT [5]. Similar success was reported in a number of subsequent series, although these were all uncontrolled studies in carefully selected patients [8]. Our results of patients with EDS/HSD are congruent with those, and we have found that most diagnoses of TCS occurring in the EDS/HSD population have a normally positioned conus, that is, above the mid-L2 vertebral level.

The surgical technique used for this series involved laminoplasty. This was accomplished with miniature titanium plates to hold the lamina in place. Demineralized bone was placed into the interstices to facilitate the fusion. The empirical reasoning behind the lamina replacement included minimization of epidural compression by scar tissue, lessening the risk of CSF leak, facilitation of reentry for repeat untethering, and reestablishment of normal bone structure and stability.

However, sectioning of the FT in the setting of occult TCS has been debated [13,16-17]. A small, randomized controlled trial found no significant difference in the improvement of urological symptoms in 10 patients with occult TCS treated surgically versus 11 treated with medical management alone [18]. This latter study did not address pain, sensory, and motor findings; however, it does draw attention to the absence of standardized urological parameters with which to study efficacy, the absence of consensus upon inclusion and exclusion criteria, and the ambiguity of language surrounding the condition of TCS [17]. This deficiency of standardized terms and language is being addressed with the development of tethered cord “common data elements”, by which future studies can more specifically measure appropriate TCS endpoints.

The co-morbid relationship between EDS and TCS remains ill-defined. TCS has been reported in patients with Chiari malformations type I (CM-I) [9,19-20]. In a large study of 2,813 subjects with CM-I, 357 (12.7%) were diagnosed with hereditary disorders of connective tissue (HDCT), predominantly hEDS [21]. In a subsequent study of the same subject population, occult TCS was identified in 14% [6]. Though the incidence of TCS appears more frequent in the EDS population [7], epidemiological evidence is still lacking.

There remains controversy over the pathophysiology of TCS [17-18]. Early work in which tension was applied to the filum terminale of anesthetized cats demonstrated that increasing tension on the filum resulted in an increased weakening of the hind limbs and urinary incontinence. Spinal cord traction was shown to be linked to electrical suppression, cytochrome reduction, oxygen and glucose consumption, and hypoxemia. Mild and moderate cord traction resulted in reversible changes, whereas severe traction produced irreversible changes [3]. Though tension on the spinal cord was initially linked with a thickened filum or elongated cord, Yamada reported on subjects in whom the spinal cord was not elongated: 28% of his subjects with TCS showed neither cord elongation nor a thickened terminal filum [3-4]. Subsequent histological and biomechanical studies showed that the filum of the TCS patient was less elastic than that of the non-TCS patient. Yamada concluded that loss of viscoelasticity was the main predisposing factor for the development of TCS [3]. This work thus prompted the sectioning of the filum terminale, to release tension upon the conus medullaris [10]. More recently, the importance of loss of elasticity of the filum in TCS has been confirmed by Klinge et al, who demonstrated in patients with hEDS and TCS that the filum became inelastic at 4% strain (increase in length of 4% over original length), as opposed to the filum of non-EDS patients, in whom the elasticity was lost at a strain of 8% [11]. We concur with Yamada and Klinge, and add that increased bending of the spine, due to pathologic flexibility of the spine in EDS, results in increased intermittent biomechanical stretching (strain) imposed upon the spinal cord by the relatively inelastic filum terminale. 

This pilot study showed a statistically significant improvement in pain, ability to walk, and urinary symptoms. Patient satisfaction was also high, and there was sufficient symptomatic improvement for most patients to state that surgery was warranted. There was no difference in outcomes between those patients diagnosed with the conus at a normal position (occult TCS) and those identiﬁed on imaging with a low-lying conus. The successful outcomes after sectioning of the ﬁlum terminale in subjects with a conus in the normal position suggest that the MRI ﬁndings have uncertain signiﬁcance in terms of making the diagnosis of TCS, and of predicting the outcome of tethered cord release in this patient population.

Limitations

The positive results of this study should be considered with caution. The study is retrospective, constrained by the nature of preoperative data collection, recall bias, short follow-up duration, and lack of validated outcome measures, and is compromised by the poor return of questionnaires. Moreover, despite excellent recent experimental data, the underlying pathophysiology remains controversial. Although the response rate was similar to other survey studies, there may be a skewed study population, such that those who did not respond to mail-in surveys were doing well and not in need of further care from their neurosurgeon. Moreover, a number of patients did not have responses to some variables, for example, only 19 patients reported both pre and postoperative walking distances.

## Conclusions

This pilot study supports the hypothesis that sectioning of the ﬁlum terminale for TCS in the patient population diagnosed with EDS/HSD is reasonably safe, with a low complication rate, one patient having suffered a CSF leak postoperatively. Patients reported a high satisfaction rate with significant improvement in low back pain, tripping, leg weakness, pain climbing steps, sensory loss in the lower extremities, and urinary incontinence, urgency, and frequency. The authors caution, however, that there may be serious complications of the surgery, and that patients should undergo a rigorous and reasonable screening process before embarking upon surgery. Despite the limitations of the study, the data support a consideration of a prospective, randomized, multicenter, clinical trial to assess the appropriate diagnosis of TCS and the eﬃcacy of sectioning of the FT in the population of patients with EDS.
